# Differences in Spontaneous Interactions of Autistic Children in an Interaction With an Adult and Humanoid Robot

**DOI:** 10.3389/frobt.2020.00028

**Published:** 2020-03-05

**Authors:** Bob R. Schadenberg, Dennis Reidsma, Dirk K. J. Heylen, Vanessa Evers

**Affiliations:** ^1^Human Media Interaction, University of Twente, Enschede, Netherlands; ^2^Institute of Science and Technology for Humanity, Nanyang Technological University, Singapore, Singapore

**Keywords:** autism spectrum condition, child-robot interaction, descriptive study, interaction types, individual characteristics

## Abstract

Robots are promising tools for promoting engagement of autistic children in interventions and thereby increasing the amount of learning opportunities. However, designing deliberate robot behavior aimed at engaging autistic children remains challenging. Our current understanding of what interactions with a robot, or facilitated by a robot, are particularly motivating to autistic children is limited to qualitative reports with small sample sizes. Translating insights from these reports to design is difficult due to the large individual differences among autistic children in their needs, interests, and abilities. To address these issues, we conducted a descriptive study and report on an analysis of how 31 autistic children spontaneously interacted with a humanoid robot and an adult within the context of a robot-assisted intervention, as well as which individual characteristics were associated with the observed interactions. For this analysis, we used video recordings of autistic children engaged in a robot-assisted intervention that were recorded as part of the DE-ENIGMA database. The results showed that the autistic children frequently engaged in exploratory and functional interactions with the robot spontaneously, as well as in interactions with the adult that were elicited by the robot. In particular, we observed autistic children frequently initiating interactions aimed at making the robot do a certain action. Autistic children with stronger language ability, social functioning, and fewer autism spectrum-related symptoms, initiated more functional interactions with the robot and more robot-elicited interactions with the adult. We conclude that the children's individual characteristics, in particular the child's language ability, can be indicative of which types of interaction they are more likely to find interesting. Taking these into account for the design of deliberate robot behavior, coupled with providing more autonomy over the robot's behavior to the autistic children, appears promising for promoting engagement and facilitating more learning opportunities.

## 1. Introduction

Autism Spectrum Condition (ASC) is a lifelong neurodevelopmental condition that affects the way an individual interacts with others and experiences the surrounding world. According to the DSM-V (American Psychiatric Association, 2013), diagnostic criteria for ASC include two core features, namely (a) difficulties in social interaction and communication, and (b) the presence of rigid and repetitive patterns of behaviors and limited personal interests. Current prevalence estimates of ASC found that around 1 in every 100 individuals is on the autism spectrum (Brugha et al., 2011; Elsabbagh et al., 2012), many of whom struggle to find and retain employment, to live independently, and to sustain friendships and intimate relationships (Howlin et al., 2004). For Europe, this means ~7 million autistic individuals. If you include their families, ASC is a part of daily life for more than 24 million individuals.

To support autistic individuals in living a life of their own choosing, interventions have been developed that aim to teach various social, cognitive, and behavioral skills. In recent decades, researchers are studying whether such interventions can be enhanced through the use of robots. Studies on such robot-assisted interventions for autistic children often report that the robot has a positive effect on the child's engagement (Scassellati et al., 2012; Simut et al., 2016). In turn, this can improve learning gains, as engagement is considered to be a necessary prerequisite for learning (McCormick et al., 1998), where higher engagement results in more opportunities for cognitive and social skill learning (Greenwood, 1991; Fredricks et al., 2004), as well as fewer disruptive behaviors used by the autistic child to avoid or escape the task (Gunter et al., 1993). Next to having a positive effect on engagement, robots are also thought to be less complex in terms of perceptual processing, where a robot's behavior does not have the richness of social cues of human behavior (Sartorato et al., 2017). Robots could also deliver “on demand” social skill learning, and provide quantified metrics of the child that can be used by an adult to further tailor the learning content to the child (Scassellati, 2007). In all these projects, researchers leverage well-designed interactive tasks and fitting robot behaviors to achieve impact with their robot-assisted intervention.

Despite these promising findings, achieving sustained engagement in a robot-assisted intervention that can lead to learning remains challenging. While studies on robots for autistic children generally report a positive effect of the robot on engagement, they also report on children who show very low levels of engagement or are not engaged (Rudovic et al., 2017; Desideri et al., 2018), or quickly lose attention within a session (Tapus et al., 2012). Moreover, sustaining high levels of engagement over multiple sessions is difficult (Desideri et al., 2018), where initially engaging interactions can become boring and too repetitive over time (Srinivasan and Bhat, 2013). Additionally, some interactions may be very rewarding to the child and keep them engaged, but do not facilitate learning a targeted skill. Indeed, even when the children are engaged, current robot-assisted interventions do not necessarily lead to learning (Tapus et al., 2012; Kim et al., 2013; Pop et al., 2014; Simut et al., 2016; Desideri et al., 2018).

In part, these issues can be explained by individual differences among autistic children in what they do and do not consider interesting. In addition to this natural variation in interests, autistic children are also disparate in their abilities and needs. Autism is a spectrum condition, which means that while all autistic individuals share the two core features of ASC, the manifestation and severity of these features differs *widely* among individuals. In terms of cognitive functioning, some autistic individuals have severe intellectual disability while others have exceptional intelligence (Grzadzinski et al., 2013). Also, language ability is highly variable in ASC, ranging from individuals who never develop spoken language to those with intact spoken language, but with difficulties in the pragmatic use of language. Next to difficulties, autistic individuals can also show areas of strength, such as in visuo-spatial skills (Shah and Frith, 1983), memory (Plaisted et al., 1998), or musical ability (Heaton, 2003). Altogether, a personalized approach to design of robot-assisted intervention is essential for autistic children to engage in it and learn; the robot's behavior needs to be in line with the interests, needs, and abilities of the child.

To design deliberate robot behavior, aimed at engaging autistic children and facilitating learning, we then need to understand what interactions are interesting to them and how their individual characteristics play a role in this. There is a lot of work that provide qualitative descriptions of how autistic children interact with a specific robot (e.g., Feil-Seifer and Matarić, 2009; Kozima et al., 2009; Robins et al., 2009; Tapus et al., 2012; Costa et al., 2015; Boccanfuso et al., 2016) which can provide some guidance in the design of a robot-assisted intervention. For instance, in their study with the machine-like robot Sphero, which looks like a billiard ball, Boccanfuso et al. (2016) observed the responses of autistic children in relation to the robot's various expressions of emotions. The robot's behavior was limited to rolling as a way to move, using its LED's to change color, and playing music. Responses varied from pushing, kicking, dropping, holding, and picking up the robot. Tapus et al. (2012) used the humanoid robot NAO and reported a detailed description of the interactions of four autistic children. Spontaneous interactions between the child and robot were observed, such as the child touching the robot. NAO also elicited interactions between the child and the experimenter, where children requested certain robot behaviors, or shared their enjoyment with the experimenter. We consider spontaneous behaviors, such as those reported above, to be indicative of potential for engagement. The behaviors are spontaneous, which means that there is no observable prompt that led to the behavior (contrary to responsive behaviors which require a prompt), which can indicate that the children are intrinsically motivated to engage in such behavior (Deci and Ryan, 1985). That is, they are motivated to perform the behavior for its inherent satisfaction rather than for some separable consequence (Ryan and Deci, 2000). In turn, designing deliberate robot behavior to support intrinsically motivating interactions may be particularly promising in keeping them engaged. However, our current understanding is insufficient to translate reported insights into design. Studies reporting on specific autistic child-robot interactions generally have <10 participants (Begum et al., 2016), and are qualitatively rich, but largely not quantitative. Moreover, participant characteristics are often not, or insufficiently, reported in studies on robots for autistic children (Diehl et al., 2012; Begum et al., 2016). This makes it difficult to ascertain the abilities and difficulties of the participants and generalize findings to more specific subgroups of autistic children to whom we can tailor the robot's behavior.

To conclude, robots are promising tools to enhance interventions for autistic children, and specifically also to promote the engagement of autistic children in interventions, creating more learning opportunities for the children. However, it is difficult to translate this general insight to design of actual deliberate robot behavior, because our understanding of autistic child-robot interaction is currently too limited. We do not know what child-robot interactions are particularly engaging to autistic children. Moreover, there are large individual differences among autistic children in their needs, interests, and cognitive capabilities, which need to be taken into account during the design of the robot's behavior.

In this descriptive study, we report on an analysis on the behavior of autistic children in the context of a robot-assisted intervention featuring a robot-assisted intervention. The interactions were collected as part of the DE-ENIGMA database^1^, which hosts the largest set of recordings to date of autistic children (*N* = 121) engaged in a robot-assisted intervention featuring a humanoid robot. Specifically, the analysis reports on interactions that were *spontaneously* initiated by the children. We aim to address the following research questions:

1. What interactions did the autistic children spontaneously engage in within the DE-ENIGMA robot-assisted intervention?
2. Which individual characteristics predicted these various interactions?

Specifically, we analyzed both child interactions with the robot and interactions with the adult that made a reference to the robot in some way. To investigate whether certain interactions are of interest to particular groups of autistic children, we used the scores of various ASC diagnostic assessments and demographics to differentiate among children. By answering the above two research questions, we hope to deliver concrete insight into what robot behaviors to design for engaging certain autistic children in a robot-assisted intervention.

The remainder of this article is structured as follows. Section 2 reviews related work on how autistic children interact with objects and with robots. These works form the basis of the coding scheme that we used to annotate the videos. Then, we report on the methods that we used and describe the DE-ENIGMA robot-assisted intervention in section 3. In section 4, we present a description of our sample of the data collection and the results of the analysis on the children's behavior. We conclude the article with a discussion on how we interpret the observed interactions within the DE-ENIGMA robot-assisted intervention in section 5 and conclude on our research questions in section 6.

## 2. Background

### 2.1. Interactions With Robots

The type of robots that are used in robot-assisted interventions are referred to as socially assistive robots (SAR). The main feature of these robots is that they interact socially with the user as a means of helping them in some way (Feil-Seifer and Matarić, 2011b). What this interaction looks like, and thus what the design of the robot's behavior should try to achieve, depends on how the robot is positioned within an intervention. In a review, Diehl et al. (2012) identified three types of SAR applications in interventions for autistic children. Firstly, the robot can be used to elicit a target behavior. This can then create a situation that can be utilized by an adult—or the robot—to promote prosocial behavior. An example of this application is the intervention described by David et al. (2018), where the robot tries to elicit joint attention and provides feedback. Secondly, the robot can be used as a tool for learning and practicing a target behavior. For instance, in Chevalier et al. (2017), the robot mimics the child's facial expressions and serves as a mirror for the child to enable playful practice with facial expressions. In the intervention, the robot is a tool used by the adult who asks the child to make specific facial expressions of emotion. The resulting situation can then be utilized by the adult to teach more about the recognition and expression of that emotion. Lastly, the robot can provide encouragement and promote interaction with another person. An example of this approach is the intervention reported by Huskens et al. (2015), where the robot encouraged an autistic child and that child's sibling to cooperate with each other in a Lego construction task.

From a pedagogical point of view, it does not necessarily matter whether the child interacts with the robot directly or whether the robot promotes interaction between the child and another person, as learning can occur in either case. For example, teaching joint attention can be done through using the robot as an object of shared attention between adult and child (e.g., Robins et al., 2004), or the robot itself could direct the child's attention elsewhere by saying “Look!” and pointing (e.g., David et al., 2018). While many robot-assisted interventions seek to actively design robot behaviors for promoting interaction between the child and another person, there is a plethora of studies reporting that these interactions also occur spontaneously (Robins et al., 2004, 2005; Duquette et al., 2008; Feil-Seifer and Matarić, 2009; Kozima et al., 2009; Kim et al., 2013; Costa et al., 2015). For instance, Kozima et al. (2009) reported autistic children turning to the adult and sharing their enjoyment after the robot responded unexpectedly to the child's touch. When the child initiates such a behavior, it is commonly referred to as a *social overture* (e.g., Lord et al., 2012), which is a behavior whose purpose is to communicate social intent. Teaching autistic children to spontaneously initiate interactions with others can also be a goal on its own (e.g., in Pivotal Reponse Treatment, Koegel and Koegel, 2006). Autistic children often have difficulty initiating social interactions (Stone et al., 1997; Koegel et al., 1999b), which may limit their ability of self-learning (Koegel et al., 1999a) and eliciting teaching interactions from their environment (Koegel et al., 2003). For this reason, researchers are also looking into enhancing interventions aimed at promoting social initiation skills with a robot (Huskens et al., 2013).

The above outlines the various kinds of social interactions between autistic child, robot, and other persons, robot-assisted interventions aim to achieve. However, from the child's perspective, the interaction “intended” by the designers may not be of interest or even be a logical response to the robot's morphological and behavioral cues. How these morphological and behavioral cues of a robot are processed depends on the child's cognitive ability (Johnson, 2003). Given that ASC affects the cognitive development, these cues can be process very differently from one autistic child to another. One may interpret these cues and consider the robot to be a *social actor*, whereas another child may come to the conclusion that the robot is an *inanimate non-social object*. As a result, there are large individual differences in how autistic children interact with a robot. While the cognitive processes underlying the perception of robots by autistic children remains unclear, there is behavioral evidence of autistic children interacting with robots in object-like manners, as well as interactions where they may consider the robot a social actor (Short et al., 2017). Moreover, compared to typically developing children who readily attribute human-like characteristics to a robot (Beran et al., 2011), there is preliminary evidence for autistic users, where this tendency was found to be reduced for autistic children (Chaminade et al., 2015) as well as autistic adults (Bird et al., 2007). Understanding how individual characteristics impact the interaction between an autistic child and robot is essential to effectively designing robot behavior to engage these children and in choosing the robot morphology that is best suited to facilitate these robot behaviors.

Thus, while robot-assisted interventions are typically designed to elicit social interaction, this does not mean that autistic children also consider the robot to be a *social actor*. Because we can also expect object-like interactions with a robot, we will briefly discuss related work on how autistic children interact with objects.

### 2.2. Interaction With Objects

While robots are relatively novel technology that autistic children interact with, their interactions with regular objects—such as toys—have been studied extensively. Much of the research on how autistic children interact with objects is conducted in a play setting, where researchers study how the complexity of play develops as the development of the child progresses. In particular, the children's ability to focus their attention, motivation, and representational capacities, play an important role in their interaction with an object (Vig, 2007; Lifter et al., 2011). Different developmental stages of play can be distinguished, which generally include sensorimotor or *exploratory play, relational play*, conventional or *functional play*, and representational or *symbolic play* (Libby et al., 1998; Casby, 2003; Naber et al., 2008). Children start out with exploratory play with objects and gradually develop the ability to create cognitive representations of objects and events required for more sophisticated types of play, such as symbolic play (Stagnitti, 2004). These developmental stages are not mutually exclusive, and children can exhibit a variety of play types. Autistic children seem to follow the same developmental trajectory of the play types as typically developing children (Vig, 2007), but may show the less sophisticated play types due to developmental delays.

*Exploratory play* is the earliest type of interaction with objects, starting to emerge at around 3–4 months of age during typical development, and are marked by oral or manual manipulation of objects, such as spinning, smelling, or mouthing (Williams, 2003). Through this type of play, children learn about the properties of different objects and how they relate to the world around them. As children begin to understand how objects relate to each other, they start showing *relational play*. This is play where a child uses different objects and relates them to each other in a way that does not indicate functional use of the object (i.e., using an object for its intended purpose). For example, nesting one object in another, or stacking objects. When children become aware of and show attention to the different properties of objects and their uses, objects start to be used in a conventional manner, which is called *functional play*. For example, children may push a toy car, or put a telephone to their ear. This type of play requires a first-order representation of the object. As children start to develop the cognitive capacity for second-order—or meta—representations of objects, they gain the ability to decouple mental representations of objects from reality (Leslie, 1987). Objects can then be used by pretending it is something else, attributing false properties to the object (e.g., the robot is ill), or referencing to an object as if it were present. This is called *symbolic play*.

Compared to typically developing children, or children with other developmental difficulties, the manifestation and frequency of the play types for autistic infants (Naber et al., 2008; Pierucci et al., 2015) and autistic children (Libby et al., 1998; Wilson et al., 2017) is markedly different. During exploratory play, autistic children seem to prefer using proximal senses of touch, taste, and smell to explore objects, rather than using vision (Williams, 2003; Naber et al., 2008). When they do visually inspect an object, they may place the objects close to their eyes, or they may focus on one aspect for an extended period of time (Williams, 2003). Libby et al. (1998) reported observing fewer instances of relational play in autistic children than typically developing or children with Down syndrome. Similarly, autistic children engage less frequently in functional play compared to other children (Williams et al., 2001; Christensen et al., 2010). Furthermore, their functional play tends to be less varied, integrated, and complex than that of other children (Williams et al., 2001; Christensen et al., 2010). Symbolic play is the play type with which most autistic children have difficulty (Jarrold, 2003). According to Jarrold (2003), autistic children may have the underlying capacity to understand symbolic play, but are less inclined to spontaneously engage in symbolic play. One explanation for this is that autistic children are more tied to the properties of an object and may have difficulty overriding these properties by pretending it is something it is not.

In summary, the four play types describe how children may engage with an object. When applied to the interaction with a robot, child-robot interactions where the child explores the robot's materials through any of the senses would classify as an exploratory interaction. Relational interaction with the robot are interactions where the child uses additional objects with the robot in a non-functional manner. Functional interactions are interactions for which the robot was designed. In most cases of robot-assisted interventions, these would be social interactions. The distinction between functional and symbolic interaction is more delicate when it comes to SAR, as they are designed to elicit social interaction. This is only possible when the robot is viewed as a *social actor* to a certain extent, rather than a bunch material wired together to form a *non-social object*. Therefore, the appearance of robot and their behavior purposefully create the illusion of animacy (Castro-González et al., 2016). Attributing animacy to a robot can then be considered a false belief. Whether the autistic child-robot interaction stems from the child's belief that the robot is a living entity, or merely because they learnt that this is how you should interact with a robot (e.g., by observing an adult interacting with the robot) is difficult to determine. Before an interaction can be classified as symbolic, there needs to be clear evidence that the child is aware of attributing a non-existing property to an object (Lillard, 2001). In the remainder of this article, we will therefore not make the distinction between functional and symbolic interactions with the robot.

## 3. Materials and Methods

### 3.1. Dataset

#### 3.1.1. DE-ENIGMA Database

The work described in this article is part of the DE-ENIGMA project, in which we participated in the collection of audio and video recordings to develop a publicly available multi-modal database of autistic children's interactions—the DE-ENIGMA database. In this article, we only present an analysis that we conducted on the data that were collected for this database, along with a small summary on the human-robot interaction recorded in the database. A detailed description on the DE-ENIGMA database itself is reported elsewhere.

The data collection for DE-ENIGMA involved recording an intervention for autistic children that was either robot-assisted or adult-only. The children participated in only one of these two conditions. The recordings took place in either Serbia or the United Kingdom. The children were recruited from three special education settings in the United Kingdom, which were also the locations of where the sessions took place. All of the children had received an independent clinical diagnosis of ASC according to criteria of the ICD-10 (World Health Organization, 1992), DSM-IV-TR (American Psychiatric Association, 2000), or DSM-V (American Psychiatric Association, 2013). The majority of the autistic children had additional intellectual disabilities and language challenges.

Ethical approval for the data collection in the United Kingdom for the DE-ENIGMA database was reviewed and approved by the ethics committee of the University College London, Institute of Education, and is registered under reference number “REC 796.” For some of the autistic children who participated, the parents only granted consent for using their child's data within the project, and not for inclusion in the publicly available database.

#### 3.1.2. Data Selection

**Figure 1:**
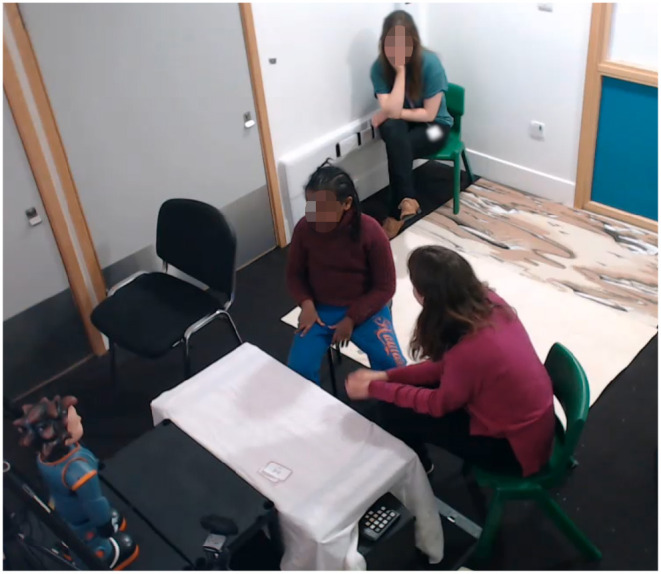
Screenshot from one the sessions in the United Kingdom, where the adult is using the robot for the DE-ENIGMA learning task. The adult at the back is a school staff member who accompanies the child.

The video recordings used for the analyses presented in this article are a *subset* of the video recordings that were collected for the DE-ENIGMA database. The subset of the video recordings used of our analyses only includes the recordings of the autistic children from the *United Kingdom* who participated in the *robot-assisted condition* (see Figure 1 for a screenshot of one of the video recordings). Video recordings from Serbia were excluded from our analyses to reduce the impact of different cultures on how the children interacted with the robot. Additionally, our subset includes the data of three autistic children for whom the parents only granted consent for their data to be used within the project and are not included of the database. Furthermore, our analysis uses standardized (diagnostic) tests that were collected, but could not be included in the database due to the ethical constraints; they can only be used within the DE-ENIGMA project.

The children interacted with the robot for several sessions every or every other day. The number of sessions for the children depended on their availability and progression through the learning content. For each child, one of their sessions was randomly selected for the analysis. A description of the children included in our sample is reported in section 4.1.

### 3.2. Robotic System

**Figure 2:**
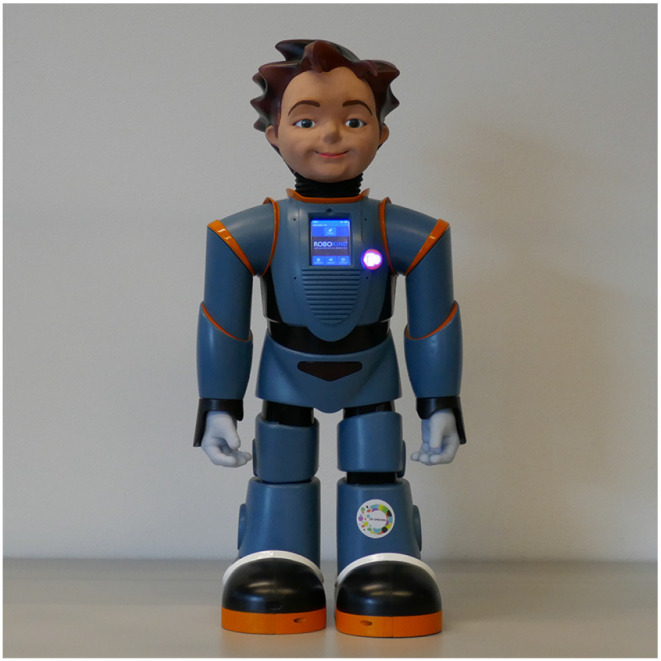
Robokind's R25 humanoid robot Zeno used in the DE-ENIGMA database recordings.

The robot used in the DE-ENIGMA database recordings is Robokind's R25 humanoid robot called “Zeno” or “Milo” (see Figure 2). The main feature of this robot is its expressive face, which can be used to display facial expressions of emotion. It has five degrees of freedom in its face, and two in its neck. The robot-assisted intervention used the default facial expressions designed by Robokind, augmented with affect bursts as described in Schadenberg et al. (2018). Additionally, Zeno had several gestures which could be used to (attempt to) elicit joint attention, various behaviors to draw the attention or to reward the child, and behavior for saying “hi” and “goodbye.” When idle, Zeno showed life-like behavior by moving its wrists, blinking its eyes, and turning its head every now and then.

The Wizard-of-Oz paradigm was used for operating Zeno, where the adult who was giving the intervention also controlled the robot. The Wizard-of-Oz interface was a small keypad that was hidden underneath the table on which Zeno was standing. A cloth over the table further obscured the keypad from the child's sight. The keypad contained controls for all of the robot behaviors, except the life-like behavior which ran autonomously.

### 3.3. DE-ENIGMA Learning Task

The robot-assisted intervention used in the DE-ENIGMA database is based on the teaching programme developed by Howlin et al. (1999) and adapted to incorporate the robot. Howlin et al.'s teaching programme focuses on teaching perception, expression, understanding, and social imagination related to the affective states happiness, sadness, anger, and fear. Prior to attending the first session, the autistic children received a social story describing the session, which included specific information about what to expect (e.g., who they would see, what they would do). This helped the children prepare for what would otherwise have been an unfamiliar situation.

The DE-ENIGMA sessions took place at the child's special education school in a separate room that was available for all sessions. The children were accompanied by their school staff member, whose main role was to provide additional support if needed but not to participate in the teaching. Each child started the first session with a free-play activity with toys to help them become comfortable with the unfamiliar adult and setting. The interaction with the robot started with a brief introduction to Zeno, where the adult would display the various behaviors of Zeno to familiarize the child with the robot. Next, the adult would demonstrate each of Zeno's dynamic facial expressions of emotion and label them. The children were then guided to work through an adapted version of the six steps defined by Howlin et al. (1999):

1. *Recognizing static emotional expressions:* recognizing Zeno's emotional facial expressions as depicted on laminated cards.
2. *Recognizing abstract static emotional expressions:* recognizing emotions expressed by emoticons on laminated cards.
3. *Recognizing dynamic emotional expressions:* matching Zeno's dynamic emotional facial expressions with static emoticons on laminated cards.
4. *Recognizing dynamic emotional expressions:* similar to step 3, but the child was also asked to express the shown emotion.
5. *Recognizing dynamic emotional expressions:* similar to step 3, but the child was also asked to express the same emotion as the robot (no label of the emotion was provided).
6. *Recognizing situation-based emotions:* recognizing how the character (the robot, the child, or another child) in a social story feels. The social stories start out as simple situation-based stories, and gradually move toward incorporating desires and beliefs.

These steps were adapted to incorporate Zeno in the following way: instead of photographs of real people for step 1, photographs of Zeno were used; and instead of the adult showing dynamic facial expressions for step 3–6, Zeno was dynamically animated to display these expressions with its face. In each of the steps, Zeno expressed the emotions and provided positive feedback (either for the correct answer, or for the child's effort). At the end of the interaction with the robot, Zeno would say “goodbye” to the child.

The children engaged in the robot-assisted intervention for several sessions, scheduled over multiple days. The exact number and length of the sessions depended on the child's progress through the intervention, and additional factors, such as their attention span. The intervention described above varied somewhat among the children, as the learning content was adapted to the child's behavior and language by the adult. The children were finished with the intervention when they had completed all six steps, or when they were not able to successfully complete a step after three separate attempts.

### 3.4. Coding Scheme

For the analysis, we observed the interactions of the autistic children during the sessions with the robot. These were then annotated with the ELAN transcription software^2^, developed by the Max Planck Institute for Psycholinguistics in Nijmegen, the Netherlands.

We annotated interactions that met the following two criteria: firstly, the interaction should be *spontaneous*, which means that the child initiated the interaction without a prompt from the adult. For example, the child could ask the robot a question, or reach for the robot to explore its physical properties. Any responses or answers given by the children in response to a specific prompt by the adult were excluded. For example, the children were frequently required to choose an emotion they recognized, and they learned this through prior prompts by the adult. Secondly, the interaction should be *directed* toward either the robot, or the adult. For the latter, the interaction should also be related to the robot in some form (i.e., the behavior would not have occurred if no robot was present). For example, asking the adult a question about the robot. Any spontaneous interactions directed toward the school staff member were not annotated, as they were asked not to be actively involved in the intervention. Potentially relevant interactions can occur at any point during the session, not only during the child-robot interaction (Dickerson et al., 2013). We therefore annotated the whole recording, which included parts where the robot was still covered by a blanket, as well as when the adult and child were engaged in free-play.

We used a grounded theory approach to design a coding scheme with which we analyzed the observed child behaviors (Saldaña, 2015). The coding scheme that we used for the analysis to annotate the spontaneous interactions of the children can be seen in Table 1. All observations were placed in a behavioral unit, which describes how the interaction manifested itself. In turn, each behavioral unit is part of a categorical unit (in bold in Table 1), which describes what type of interaction the manifestation belongs to. For the robot directed interactions, the categorical units were based on the developmental types of object play (Libby et al., 1998; Casby, 2003; Naber et al., 2008). They include exploratory, relational, and functional/symbolic interactions. We combined functional and symbolic interactions into one categorical unit because the distinction between the two is precarious when it comes to robot (see section 2.2). The exploratory interactions with the robot were restricted to some extent, due to the fragility of the robot. Beyond gently touching and visually inspecting the robot, other tactile interactions were actively prevented by the adult and the school staff member. These include possible exploratory interactions, such as banging the robot on the table, or mouthing the robot. In addition to the child interactions with the robot, we also annotated any the children's interactions toward the adult which related to the robot in some way. Such interactions were categorized as social overtures—a spontaneous social initiation of the child toward the adult.

**Table 1:** Description of the coding scheme for annotating the autistic child's interactions with the robot and robot-mediated interactions with the adult.

**Interaction type**	**Description and examples**
**Exploratory interactions with the robot**	
Touching the robot	Touching part of the robot. This also includes attempts at touching Zeno, as children were prevented from actually touching it. An attempt is classified as moving toward Zeno and reaching for it, or when the intent is clear from previous attempts at touching Zeno.
Explicit visual inspection of the robot	Moving closer to the robot, or leaning over the table, and visually inspecting the robot.
**Relational interactions with the robot**	
Using additional objects with the robot	Relating one or more objects to the robot in a way that does not indicate functional or symbolic play. For instance, putting a laminated card on top of the robot or in its mouth.
**Functional interactions with the robot**	
Imitation of the robot	Child imitates Zeno's behavior or the sounds it makes without being prompted. This excludes echolalic responses. For instance, imitating the robot's waving gesture.
Joint attention initiated by the robot	Gaze shift to area where Zeno is looking/pointing at and is saying “look.”
Talking to the robot	Any questions, comments or vocalizations directed at Zeno. For instance, asking the robot about its favorite food.
Physical behaviors directed at the robot	Physical interactions with Zeno, which are not exploring behaviors. For instance pushing Zeno, blowing air in Zeno's face, or dancing with Zeno.
Controlling the robot	Making the robot do a certain behavior through the Wizard-of-Oz keypad that controls the robot. This includes reaching for, or pressing, controls on the keypad.
**Social overtures directed at the adult and related to the robot**	
Social reference	A social reference refers to utilizing the adult's interpretation of a novel situation to formulate one's own interpretation (Feinman, 1982). For instance, looking at the adult after Zeno did something unexpected.
Shared enjoyment	Indicating and communicating pleasure to the adult regarding something the robot did. For instance, looking and smiling at adult after Zeno finished its dance.
Directing attention	Getting the adult to direct her attention to Zeno. For example, the child may be pointing toward Zeno and saying “look.”
Helping	Helping the adult with something that has to do with Zeno. For instance, covering or uncovering Zeno with a blanket.
Requesting	(non) Verbally requesting the adult to have Zeno do certain behaviors. For example, the child could ask the adult to see the robot's happy facial expression by saying “do happy,” or point to a laminated card that shows a certain facial expression of Zeno.
Conversing with the adult	Talking with the adult about the robot. For instance, asking questions about Zeno, commenting on certain features of Zeno.

### 3.5. Annotation Procedure

For all observations, we annotated the start and end time of the observations. Observations that occurred within 2 s of each other were considered as the same observation. A single main coder annotated all the recordings. To calculate the reliability of these annotations, a second coder annotated a random selection of 20% of the recordings, which contained to 19% of the annotations of the first coder. There were 21 segments in the recordings that were annotated by both coders. To determine the agreement between the two coders, Cohen's κ statistic was used. The agreement between the two coders on the behavioral units was good (Cohen's κ = 0.83, 95% CI [0.66, 1.00], *p* < 0.001). However, there was a difference in sensitivity between the two coders, as an additional 24 segments were annotated by only one of the two coders. Of these additional segments, 21 were coded only by the main coder and 3 were coded only by the second coder.

As an additional check, the second coder annotated the 21 segments that were only annotated by the first coder. For these additional observations, the second coder also had to judge whether the child's behavior was “spontaneous,” “might have been spontaneous,” or “was not spontaneous.” The reason for the additional annotation of these 21 segments was given afterwards. Of the 21 additional segments, 18 were judged to be “spontaneous,” 3 as “maybe spontaneous,” and none as “was not spontaneous.” The second coder further mentioned that the child's behavior in these segments were more subtle than in the segments that both coders initially annotated. For the additional segments, agreement was good (Cohen's κ = 0.72, 95% CI [0.51, 0.93], *p* < 0.001); the aggregated Cohen's κ over all 42 segments is 0.78 (95% CI [0.65, 0.92], *p* < 0.001), which we consider to be sufficiently high to proceed with.

We conclude that there was only a difference in sensitivity between the coders, where the main coder was more sensitive than the second coder, and not in differences in labeling segments. Both coders agreed that most of the additional segments were indeed spontaneous interactions and should be annotated. Difficulties with sensitivity in the behavioral analysis of autistic children is a known and common issue. For instance, in the development of diagnostic tools for ASC, where subtle, but potentially meaningful, social communication behaviors are difficult to identify, which makes it difficult to develop a measure that is sensitive enough to account for these behaviors (Anagnostou et al., 2015; Grzadzinski et al., 2016).

We also carried out a more detailed analysis of the coder disagreements through inspection of the confusion matrices. This showed two behavioral units that deserve special mention, namely “conversing with the adult” and “requesting.” These two units had a relatively high confusion (4 out of 15 annotations), and were confused with each other. This means that we should be careful about the distinction between these two particular units. Further results presented in this article are based on the annotations of the main coder.

### 3.6. Individual Characteristic Measures

To investigate which individual characteristics were associated with the observed interactions, we used three diagnostic measures and child demographics. These measures were collected for the children who participated in the recording session for the DE-ENIGMA database, but are not publicly available as part of the database as it is released.

#### 3.6.1. ADOS-2

The Autism Diagnostic Observation Schedule—second edition (ADOS-2, Lord et al., 2012) is a structured play session conducted by a professional, and was administered to assess the level of autism spectrum-related symptoms. Each child is given one of five modules, each with their own activities for the play session. The module is primarily selected on the basis of the child's expressive language capabilities and secondarily on the child's chronological age. Module 1 is used for children older than 31 months who do not consistently use phrase speech. To account for the differences in cognitive and adaptive functioning (Bal et al., 2016), Module 1 distinguishes between two expressive language levels, namely “few to no words” (hereafter, “Module 1, FNW”) for children who used no words or fewer than five words during the ADOS administration, and “some words” (hereafter, “Module 1, SW”) for those who used more than five words up to those who used simple phrases (Gotham et al., 2007). Module 2 is used when children can use phrase speech, but are not yet verbally fluent, and Module 3 is for verbally fluent children and young adolescents. The other two modules were not applicable to our sample given the chronological age requirements.

The ADOS-2 Calibrated Severity Score (ADOS-2 CSS, Gotham et al., 2009) is the raw ADOS-2 score controlled for the chronological age and language skills. The CSS is therefore a more meaningful score for comparing scores across modules. A score of 1–2 is interpreted as minimal-to-no evidence, 3–4 as low, 5–7 as moderate, and 8–10 as high ASC symptom severity.

#### 3.6.2. CARS-2

The Childhood Autism Rating Scale—second edition (CARS-2, Schopler et al., 2010) is a 15-item autism screening and diagnostic tool and was administered to obtain a general measure of characteristics of ASC. It is completed based on direct behavior observation by a professional as well as reports from parents, teachers, or caretakers. The total score on the CARS-2 reflects the severity of autism spectrum-related symptoms with scores of 15.0–29.5 indicating minimal-to-no evidence, 30.0–36.5 is mild-to-moderate severity, and 37.0 and higher is severe ASC symptoms.

#### 3.6.3. VABS-2

The Vineland Adaptive Behavior Scales—second edition (VABS-2, Sparrow et al., 2005) is a standardized measure of an individual's adaptive behavior—the ability to undertake daily activities. Adaptive behavior is a composite of five domains, namely the communication, daily living skills, socialization, motor skills, and maladaptive behavior domains. In this article, we report on the communication and socialization domain scores. The former addresses receptive as well as expressive language usage, and the latter reflects functioning in social situations. The scores on the domains are standard scores (*M* = 100, *SD* = 15). For descriptive purposes, we also report on the Adaptive Behavior Composite, which reflects an individual's overall adaptive behavior, and is calculated using the domain scores.

#### 3.6.4. Child Demographics

For the child demographic characteristics, the children's chronological age and sex were included in the analysis.

## 4. Results

### 4.1. Description of the Sample

The sample for the analysis consisted of 31 (84% male) autistic children from the United Kingdom, between the chronological age of 5–12 years. They were randomly assigned to the robot-assisted condition for the DE-ENIGMA database recordings. Of the 31 autistic children, 28 are included in the public DE-ENIGMA database. The other three autistic children participated in the robot-assisted intervention, but did not consent for their recordings to be included in the database; they can only be used for studies within the DE-ENIGMA consortium. The average characteristics of the children can be seen in Table 2.

**Table 2:** Average characteristics of the 31 autistic children who were included in the analysis.

**Measure**	***n***	**Mean (SD)**	**Range**
Age (years:months)	31	8:4.4 (2:2.7)	5:1–12:2
Sex			
Male	26	–	–
Female	5	–	–
ADOS-2 CSS			
Module 1, FNW	10	6.60 (1.84)	3–9
Module 1, SW	14	6.29 (0.83)	5–8
Module 2	6	6.33 (1.86)	3–8
CARS-2	31	33.68 (4.52)	24.5–45.0
VABS-2			
Adaptive behavior composite	20	55.45 (8.48)	46–74
Communication domain	20	58.00 (12.55)	36–79
Socialization domain	20	55.60 (7.34)	43–68

The ADOS-2 assessment was completed for all but one of the children included in the sample. For the ADOS-2 assessment, Module 1 was used for 24 children, of which 10 used few-to-no words and 14 used some words. There were six children who had phrase speech, but were not yet verbally fluent, for whom Module 2 was used. There were no children for whom Module 3 was deemed appropriate. The child for whom there is no ADOS-2 score was unable to participate in the ADOS-2 play session as he would not engage with the examiner that conducted the assessment. The CARS-2 assessment was completed for all children. All children scored above the ASC cutoff on either the CARS-2 (30 or higher) or the ADOS-2 (4 or higher). On average, the children had moderate autism spectrum-related symptoms. The VABS-2 was assessed through survey interviews with the child's parents, and was completed for 20 children.

### 4.2. Observed Interaction Types

The analysis led to a total of 225 annotations in 450 min of video recordings. The sessions that were randomly selected for the analysis lasted from 5 min 55 s up to 37 min 12 s. On average, the sessions lasted 14 min 31 s (*SD* = 8 min 02 s). In our sample, the session ranged from the 1st to 7th sessions (*M* = 3.03, *SD* = 1.43). Twelve sessions contained the free-play activity, which lasted 6 min 27 s on average (*SD* = 3 min 05 s). Of the annotations, only seven occurred during the free play prior to the intervention with the robot, when the robot is still covered by a blanket.

**Figure 3:**
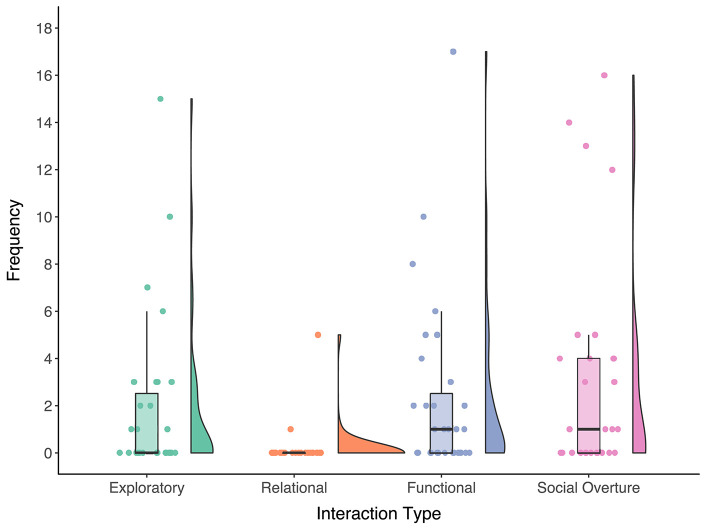
Raincloud plots (scatter, box, and density plot) that show the frequency of the interaction types observed per autistic child.

The frequency and distribution of the observed interaction types can be seen in Figure 3. Most of the spontaneous interactions with the robot classify as functional interactions (*n* = 71), and were observed for 58% of the children. Exploratory interactions with the robot (*n* = 57) were observed for 42% of the children. For two children (6%), relational interactions with the robot were observed for a total of six annotations. A total of 91 social overtures were observed, spread out over 16 children (52%).

Notably, for eight children we did not observe *any* spontaneous interactions. They were all male and had moderate to severe ASC symptoms. Of these eight children, two had an aversive reaction to the robot and left the room shortly thereafter. Both were assessed with ADOS-2 Module 1, SW. For one it was the first session, and for the other it was the second session. Four children did not seem to understand nor engage in the learning task. The adult spent most time trying to explain the task and focus the child's attention to the task. All of the four children were non-verbal. Two children engaged with the learning task and interacted with the robot and the adult when prompted, but did not initiate any interactions themselves. Both children were assessed with ADOS-2 Module 1, where one used some words and the other used few-to-none.

### 4.3. Individual Differences in Interaction Types

#### 4.3.1. Correlations Between the Individual Characteristics and Interaction Types

To calculate the association between the individual characteristics measures and interaction types we use Kendall's Tau-b partial correlation, given the large amount of tied ranks, skewed distribution, and outliers (see Figure 3). The partial correlations were controlled for differences in session length and can be seen in Table 3. The ADOS-2 Module used during the ADOS-2 assessment showed positive associations with spontaneous functional interactions (τ_*p*_ = 0.39, 95% CI [0.16, 0.58], *p* = 0.003), social overtures (τ_*p*_ = 0.63, 95% CI [0.45, 0.76], *p* < 0.001), and the total number of spontaneous interactions (τ_*p*_ = 0.41, 95% CI [0.18, 0.60], *p* = 0.002). The correlations for the communication domain score of the VABS-2 were not significant, but did show a similar trend to the ADOS-2 Module correlations. We found no evidence of an association between either the ADOS-2 Module or VABS-2 CD and spontaneous exploratory or relational interactions.

**Table 3:** Kendall's Tau-b partial correlations that show the strength of the association between the frequency of interaction types and the total number of spontaneous interactions with the children's chronological age, ADOS-2 Module, ADOS-2 Calibrated Severity Score (CSS), CARS-2 score, and the VABS-2 communication (CD) and socialization domain (SD).

	**Interaction type**				
**Measure**	**Exploratory**	**Relational**	**Functional**	**Social overture**	**Interactions total**
Age	−0.12	−0.05	−0.13	0.08	−0.11
**Language ability**					
ADOS-2 module	−0.05	0.08	0.39^**^	0.63^***^	0.41^**^
VABS-2 CD	−0.16	0.11	0.32	0.31	0.21
**Autism spectrum severity**					
ADOS-2 CSS	0.14	−0.01	−0.35^**^	−0.22	−0.19
CARS-2	0.06	−0.03	−0.18	−0.28^*^	−0.21
**Social functioning**					
VABS-2 SD	−0.06	0.02	0.33^*^	0.35^*^	0.34^*^

*The partial correlations were controlled for differences in session length*.

* *Correlation is significant at the 0.05 level (2-tailed)*.

** *Correlation is significant at the 0.01 level (2-tailed)*.

*** *Correlation is significant at the 0.001 level (2-tailed)*.

The ADOS-2 CSS was negatively associated with spontaneous functional interactions (τ_*p*_ = −0.35, 95% CI [−0.55, −0.11], *p* = 0.008), while the CARS-2 score showed a negative association with social overtures (τ_*p*_ = −0.28, 95% CI [−0.49, −0.04], *p* = 0.027). No evidence was found for either score regarding an association with exploratory or relational interactions, nor with the total number of spontaneous interactions.

The child's functioning in social situations, as measured through the VABS-2, showed a positive association with the spontaneous functional interactions (τ_*p*_ = 0.33, 95% CI [0.02, 0.58], *p* = 0.047), social overtures (τ_*p*_ = 0.35, 95% CI [0.04, 0.60], *p* = 0.036), and total number of spontaneous interactions (τ_*p*_ = 0.34, 95% CI [0.03, 0.59], *p* = 0.040).

To calculate whether the child's sex was associated with initiating different types of interactions, we used the Mann-Whitney *U* test. Being male was coded as 0 and female as 1. We found no evidence of an association between sex and exploratory interactions (U = 47.00, *p* = 0.313, *r* = 0.17), relational interactions (U = 60.00, *p* = 1.000, *r* = −0.19), functional interactions (U = 56.00, *p* = 0.636, *r* = 0.09), social overtures (U = 63.00, *p* = 0.934, *r* = −0.02), or the total number of spontaneous interactions (U = 48.00, *p* = 0.376, *r* = 0.17). To account for differences in session length, we took the number of interactions per minute.

#### 4.3.2. Novelty Effect

In our selection of the participants' video recordings, we randomly selected a session for each participant. To check whether there was a novelty effect for the interaction types, we calculated correlations between the session number and the interaction types. Given the large amount of tied ranks, skewed distribution, and outliers, we again use Kendall's Tau-b correlation. The session number showed a positive association with exploratory (τ = 0.09, 95% CI [−0.23, 0.38], *p* = 0.566), relational (τ = 0.13, 95% CI [−0.04, 0.33], *p* = 0.429), functional (τ = 0.22, 95% CI [−0.07, 0.49], *p* = 0.136), social overtures (τ = 0.26, 95% CI [−0.09, 0.55], *p* = 0.083), and total number of spontaneous interactions (τ = 0.19, 95% CI [−0.10, 0.43], *p* = 0.174). None of these correlations were statistically significant.

#### 4.3.3. Intercorrelations

The correlations between the individual characteristics can be seen in Table 4. Given the differences in type of data between the measures, we report either Kendall's Tau-b, due to small sample size and non-normality, or point-biserial correlations for sex. The likelihood ratio between sex and the ADOS-2 Module showed no significant difference [$\chi_{\left(2\right)}^{2}$ = 0.14, *p* = 0.934).

**Table 4:** Correlations between the independent variables of age, sex, ADOS-2 Module, ADOS-2 Calibrated Severity Score (CSS), CARS-2 score, and the VABS-2 communication (CD) and socialization domain (SD).

	**Age**	**Sex**	**ADOS-2 module**	**ADOS-2 CSS**	**CARS-2**	**VABS-2 CD**	**VABS-2 SD**
Age	1	−0.09	0.09	0.09	0.10	−0.21	−0.30
Sex		1	n/a	−0.20	−0.02	−0.22	0.03
ADOS-2 module			1	−0.11	−0.34^*^	0.60^**^	0.45^*^
ADOS-2 CSS				1	0.40^**^	−0.40^*^	−0.45^*^
CARS-2					1	−0.30	−0.23
VABS-2 CD						1	0.58^**^
VABS-2 SD							1

*The correlations are Kendall's Tau-b correlations, except for correlations with sex, which are point-biserial correlations*.

* *Correlation is significant at the 0.05 level (2-tailed)*.

** *Correlation is significant at the 0.01 level (2-tailed)*.

### 4.4. Manifestations of the Interaction Types

Below follows a qualitative analysis describing how the interaction types were manifested during the robot-assisted intervention and how they differed between the children. The frequencies of the manifestations for each interaction type can be seen in Table 5. The frequencies in this table are the frequencies for an average session length. We grouped the children by the ADOS-2 Module that was used, given that the children's language ability, as measured by the ADOS-2 Module, showed the strongest association with the interaction types (see previous section).

**Table 5:** Frequency and range for the interaction types and the observed manifestations, indicating how frequently certain interactions and manifestations were observed.

	**Frequency**		Number of children showing the manifestation for each ADOS-2 Module^*^ (%)		
**Interaction manifestations**	**Count**	**Range**	**Module 1, FNW**	**Module 1, SW**	**Module 2**
**Exploratory interactions**	**57**	**0–15**	**4 (40%)**	**7 (50%)**	**2 (33%)**
Touching robot	33	0–7	4 (40%)	6 (43%)	1 (17%)
Explicit visual inspection	24	0–10	2 (20%)	4 (29%)	2 (33%)
**Relational interactions**	**6**	**0–5**	**0 (0%)**	**2 (14%)**	**0 (0%)**
Using additional objects	6	0–5	0 (0%)	2 (14%)	0 (0%)
**Functional interactions**	**71**	**0–17**	**4 (40%)**	**8 (57%)**	**6 (100%)**
Controlling the robot	31	0–8	1 (10%)	4 (29%)	4 (67%)
Talking to the robot	15	0–11	0 (0%)	2 (14%)	2 (33%)
Imitation of the robot	12	0–5	2 (20%)	2 (14%)	2 (33%)
Physical behavior	8	0–3	2 (20%)	3 (21%)	0 (0%)
Joint attention	4	0–1	1 (10%)	1 (7%)	2 (33%)
**Social overtures**	**91**	**0–16**	**1 (10%)**	**9 (64%)**	**6 (100%)**
Conversing with the adult	27	0–10	0 (0%)	1 (7%)	6 (100%)
Requesting	25	0–7	1 (10%)	5 (36%)	3 (50%)
Shared enjoyment	20	0–4	1 (10%)	1 (7%)	5 (83%)
Social reference	11	0–4	0 (0%)	5 (36%)	4 (67%)
Directing attention	5	0–3	0 (0%)	1 (7%)	2 (33%)
Helping	4	0–2	1 (10%)	2 (14%)	0 (0%)
**Interactions total**	**225**	**0–48**	**6 (60%)**	**11 (79%)**	**6 (100%)**

*Given that the ADOS-2 Module showed the strongest correlation with the interaction types, this table also shows the number and percentage of autistic children with one or more observations for the interaction types and manifestations within each of the ADOS-2 Modules*.

* *One autistic child was not assessed with the ADOS-2 and is missing from these columns. We observed no spontaneous interactions for this child, who would likely have been assessed with Module 1, FNW. The values in bold are the aggregated values for the interaction types*.

#### 4.4.1. Manifestations of Exploratory Interactions

The exploratory interactions that we observed involved explicitly inspecting the robot and touching various parts of it. These two manifestations of exploratory interactions were observed for children who saw the robot for the first time as well as for children who had interacted with the robot up to six times before. Sometimes the visual inspection would precede reaching for the robot, but more often the children would immediately reach for the robot. The robot stood at the rear of a table, which prevented the children from touching the robot when they were seated. Of the children, eight stood up to get a closer look at the robot, either by leaning forward over the table, or by walking to the rear of the table and standing next to the robot. Two of them persisted in visually inspecting the robot and accounted for six and 10 annotations. Eleven children touched or attempted to touch the robot. Most of these children did so several times during a session. When the children reached for the robot, the adult and school staff member intervened, as it was often not possible to tell beforehand whether the child would gently touch the robot, or would grab the robot to explore its properties through, for example, licking, spinning, or banging the robot on the ground. The latter could potentially harm the robot.

#### 4.4.2. Manifestations of Relational Interactions

The number of other objects that could have been used in the robot-assisted intervention was limited to the learning materials and any items the children brought with them. Two children placed the laminated emotion cards in the robot's mouth after it had opened the mouth for a certain animation. When the robot closed its mouth, the card would stay clenched in the robot's mouth. One child in particular found this type of interaction interesting and accounted for five of the six observations.

#### 4.4.3. Manifestations of Functional Interactions

The manifestations of the functional interactions were grouped into five behavioral categories, namely controlling the robot through the Wizard-of-Oz keypad, imitating the robot, talking to the robot, looking at the spot where the robot pointed toward when it tried to establish joint attention, and any physical behaviors directed at the robot, such as dancing. The majority of the functional interactions with the robot involved controlling the robot through the Wizard-of-Oz keypad used by the adult. While the keypad was hidden underneath the table, nine children had found out that the robot would respond to presses on the keypad. These children would reach for the keypad several times during the session, and sometimes were successful at pressing a button which resulted in the robot performing an action.

Of the children who used semantic speech, four children spoke to the robot. This was mostly confined to saying “hello” and “goodbye” to the robot. For the three children, we had annotated their third or fourth session with the robot. One child—for whom it was the second session—accounted for most of the annotations (11 out of 15). He introduced himself to the robot, asked it several questions, and asked the robot to keep its chest light on later during the session.

The functional interaction manifestations of imitation, physical behaviors, and joint attention, are more related to the content of the DE-ENIGMA intervention. Six children spontaneously imitated the robot. These were mostly imitations of the robot's gestures and its speech (excluding children who used echolalia). One child initiated imitations of the robot's emotions during stages of the learning content where the children were not specifically asked to imitate the robot. The physical behaviors with the robot included dancing together with the robot. One child waved at the robot in response to the robot saying hello. Another child blew air at the robot's face. Notably, this child was listening to a social story where the robot was being pushed and started enacting the story by pushing the robot backwards himself. Four children followed the robot's gaze when it pointed at an empty area in the room (joint attention). One child noticed that the robot was pointing at nothing in particular, and concluded that “its in his head,” i.e., the robot imagined something.

#### 4.4.4. Manifestations of the Social Overtures

For the interactions with the adult, which were related to the robot in some form, we observed six types of interactions, namely conversing with the adult about the robot, making requests for a certain robot action, the sharing of enjoyment after the robot did something, social references after the robot did something, directing the attention of the adult to the robot, and helping the adult with the robot. Of these six types, the majority of the interactions involved requesting robot actions, the sharing of enjoyment, and for the autistic children with more expressive language, conversing with the adult about the robot.

Seven children initiated a conversation with the adult about the robot for a total of 27 observations. Prior to seeing the robot for the first time, one child seemed somewhat anxious and commented several times that the robot is not a “real robot” but a toy, and did not want to see the robot. He accounted for 10 of the 27 annotations. However, after the child calmed down and was familiarized with the robot's actions, the child showed many signs of positive affect and continued conversing and initiating conversations with the adult. For the other six children who conversed with the adult about the robot, we had annotated their second, third, or fourth session. Four children came up with a rationale to explain why the robot did a certain action. During the social stories with the robot as the main character, two children spontaneously explained why they thought the robot would feel a certain emotion. Four children had questions for the adult regarding the robot. These questions had either to do with the appearance of the robot, if the robot could do certain things, such as having dinner, or how to control the robot through the keypad. Most of the requests were verbal, although some were non-verbal, where the child would look at the adult and imitate the robot's action they wanted to see. Nine children requested to see specific robot behaviors. These requests include 12 requests for emotional facial expressions, seven to see the robot dancing, five for other gestures, and for one request it was unsure what the child was requesting.

Some of the robot's actions elicited a social response from the child in the form of sharing their enjoyment or a social reference. Of the 20 observations of shared enjoyment half are related to the child making the robot do something through the Wizard-of-Oz keypad, or doing something to the robot like putting the laminated emotion cards in the robot's mouth. The other half of the shared enjoyment observations occurred after the robot had performed one of its gestures or expressions of emotion. Social references were elicited by the robot when it did something unexpected, such as when the robot started moving for the first time or when the robot got stuck halfway through an animation, or when the child did not know how to interpret the robot's behavior. The child would then look at the adult for an explanation. The latter happened for two children when the robot tried to initiate joint attention. Rather than looking where the robot was pointing to, the children looked at the adult unsure what the robot was trying to communicate. For three children, unexpected actions of the robot also lead to them children directing the attention of the adult to the robot. Two other annotations of directing the adult's attention to the robot occurred when a child was talking with the adult about the robot, and the other when a child made the robot say “hi” using the keypad.

Lastly, we observed that four children spontaneously helped the adult to remove the blanket covering the robot at the start of the session. One child helped the adult to cover the robot with the blanket at the end of a session.

## 5. Discussion

### 5.1. Types of Sponteanous Interactions

We observed that autistic children spontaneously engage in a wide variety of interactions with the humanoid robot or the adult. In their interaction with the robot, autistic children most frequently initiated exploratory or functional interactions. Relational interactions were only observed for two children, which can be explained by the lack of other objects in the room. This made it difficult for children to engage in relational interactions.

Looking at the manifestations of the children's spontaneous interactions with the robot, it stands out that some children initiated the same spontaneous interaction many times during a session. Some children had a strong desire to touch or inspect the robot to the extent that they were preoccupied with engaging or trying to engage in such exploratory interactions. The soft, malleable material of the robot's face and hands were particularly interesting to the children. For the DE-ENIGMA robot-assisted intervention, the preoccupation with engaging in exploratory interactions was problematic, as the intervention was not designed to support learning through exploratory interaction, nor was the robot sufficiently robust that it could withstand tactile exploratory interactions for longer periods of time. The adult therefore dissuaded the children from touching the robot. Rather than preventing autistic children from engaging in exploratory interaction, it would be more motivating for them when they can learn about the targeted behavior through this type of interaction. For instance, play interactions, such as developed by Robins and Dautenhahn (2014) or Boccanfuso et al. (2016) to promote imaginary play and understand contingency and causality. In both studies, the robot facilitated touch interaction and reacted with a (affective) response. In addition to being able to learn through tactile exploratory interactions, the robot itself should also be designed to accommodate such interactions. Robots, such as Probo (Saldien et al., 2008) and KASPAR (Dautenhahn et al., 2009; Robins and Dautenhahn, 2014) have been specifically designed to accommodate tactile interaction. Such robot designs may be particularly suitable to facilitate learning for autistic children through tactile interaction. For robots that cannot withstand frequent tactile interaction, a more extensive familiarization phase that includes exploratory interaction guided by an adult might reduce the desire to explore the robot's materials in some cases.

Next to the desire to explore the robot's materials, the children were also interested in making the robot do a certain behavior. This is reflected by frequent observations of (trying to) controlling the robot through the keypad, or by requesting the behavior to the adult, as well as some children becoming preoccupied with these interactions by repeatedly initiating them. The intervention used in the DE-ENIGMA database was set up to be led by the adult, which meant that those children who repeatedly wanted to control the robot's behavior was distracting them from progressing through the learning material and mastering the targeted skill. Other studies also report frequent observations of autistic children making requests. For example, during naturalistic observations of classroom activities (Keen et al., 2002), as well as in interactions with robots (Tapus et al., 2012). Tapus et al. (2012) used the humanoid NAO robot and reported that when the child made a request and the robot conformed, the child shared his enjoyment with the experimenter. We observed similar responses to requests or when the child was allowed to control the robot. Having child-led interactions would designate the robot to a more reactive role in the interaction. This notion was also highlighted by ASC experts as an important design consideration for autistic children (Robins et al., 2007). The challenge then becomes to set boundaries and provide the context that the child can explore that aid in the learning of a targeted behavior.

In terms of learning gains, previous research in human-delivered interventions show that having an autistic child lead the interaction has mixed results. Kim and Mahoney (2004) found that the engagement improves when an adult is more responsive and less directive in a human-delivered intervention. Conversely, some autistic children may learn better with a very structured, adult-directed intervention (Kishida and Kemp, 2006). For having the child-led interactions to lead to learning depends on the child's ability to initiate and engage with the robot on their own accord, and the robot's ability to elicit such initiations. Autistic children may not necessarily be inclined to engage in social interaction, and generally have lower rates of initiation, which in turn may reduce the amount of learning opportunities (Corsello, 2005). For child-led interaction in a robot-assisted intervention, it is then pivotal to identify what factors determine whether or not an autistic child may benefit from this approach.

We also frequently observed children conversing with the robot, or conversing about the robot with the adult. However, the number of children who initiated such conversations was limited and was restricted to those that used language themselves, and one child in particular accounted for most of the talking to the robot. The observation that only a few children talked to the robot is noteworthy. The robot mostly used non-semantic speech (with the exception of the greeting and goodbye) and had an anthropomorphic design, yet the children's speech was primarily directed at the adult. While typically developing children readily make anthropomorphic inferences when interacting with a robot (Beran et al., 2011), and autistic children categorize robots very similarly to typically developing children (Peca et al., 2014), the resulting behavior of verbal autistic children is different. Possibly, our finding may be better explained by a reduced tendency of autistic children (Chaminade et al., 2015) and autistic adults (Bird et al., 2007) to attribute human-like characteristics to artificial agents; the spontaneous interactions with the robot were more akin to interactions with an object.

For several children, the robot successfully elicited social overtures of the child toward the adult, which included the sharing enjoyment with the adult, making a social reference after the robot did something that seemingly was unexpected, directing the attention of the adult to the robot, or prosocial behavior, such as helping the adult with the robot. Such interactions are not indicative of interests of the child, but instead are learning goals for the development of certain social skills that are challenging for some autistic children. An adult could exploit these interactions as an opportunity for the child to further develop this social skill.

In our study, we observed that six children did not engage with the robot or learning task. Similar to other studies that report aversive reactions of autistic children toward robots (e.g., Bekele et al., 2014; Short et al., 2017), we also observed aversive reactions of two of the six children. For one child, it was the first session with the robot. After lifting the blanket that initially covered the robot and showing the first robot behaviors, the child immediately showed signs of stress and left the room shortly after. The child showed a similar reaction in the second session, after which it was decided that there would be no third attempt. Possibly, the robot was too unfamiliar to the child, which triggered the stress response. For the other child who showed an aversive reaction, it was the second session that we annotated. Looking at the first session, the child did engage with the learning task and the robot. However, at some point, the robot's arms got stuck during one of its behaviors, which put the arms in an awkward position. The child showed a similar aversive reaction shortly after this happened. Possibly, this effect carried over to the next session. In the third (final) session, the child did not show an aversive reaction. The other four children were simply not drawn to the robot and showed little to no interaction toward the robot or the adult in the session that we annotated. After viewing their other sessions, we observed similar behavior of these children, where they also did not show any interest in the task or the robot. The severity of their autism-related symptoms was similar to the other children, however they had in common that they were all non-verbal. Possibly, autistic children with limited language ability not only initiate fewer spontaneous interactions, as aforementioned, but are also more difficult to engage in a robot-assisted intervention in general. The DE-ENIGMA robot-assisted intervention may have been too complex for these children, where a simpler interaction type may be better suited for engaging them. While we agree that autistic children react positively toward robots by and large, robots are not inherently interesting to them. This highlights the need to specifically design the robot behavior and learning task to accommodate the interests and needs of the autistic child.

### 5.2. Individual Differences in the Types of Spontaneous Interactions

For the functional interactions with the robot, we found associations with the children's language ability, severity of autism spectrum-related symptoms, and social functioning. Children with higher language ability, higher social functioning, or lower autism spectrum-related symptom severity initiated more spontaneous functional interactions with the robot. In like manner, those children initiated social overtures directed at the adult more frequently. We found no evidence of an association between any of the children's individual characteristic and the frequency of spontaneous exploratory or relational interactions. For the latter, the observed frequency is too small to meaningfully interpret the correlations. On the other hand, many of the children engaged in exploratory interactions, but the correlations with the individual characteristics were low. This could indicate that children were equally interested in these types of interactions, or possibly some other individual characteristic influences an interest in this particular type of interaction.

Language ability and autism spectrum severity were measured through two different measurement tools, but we caution for making any inferences regarding one measure being a stronger predictor than the other. Even though in our sample one measurement tool had higher correlations than the other, the measures all follow a similar trend in their association with the interaction types. Given that the number of children included in the analysis is relatively small for the type of analysis, the confidence intervals of the correlations is large. For the children's language ability and severity of autism spectrum-related symptoms, small to large correlations with functional interactions and social overtures are also reasonably compatible with our data. Therefore, we caution that one of the two measures for measuring the same construct should not be interpreted as evidence for it more strongly associated with the interaction types than the other.

In our sample, the module that was used for the ADOS-2 assessment showed the highest correlations with the interaction types. The choice of module is primarily based on the child's expressive language ability. However, note that the ADOS-2 module is not the most reliable or valid measurement of an autistic child's expressive language ability, and should therefore be carefully interpreted—more factors may, unintentionally, have been considered for assigning the modules. In studies with autistic toddlers and children, positive associations between the complexity of object play and language ability have often been reported (Mundy et al., 1987; Jarrold et al., 1993; Toth et al., 2006; Thiemann-Bourque et al., 2012). However, these positive associations are not always found (Lewis, 2003; Kang et al., 2016). Kang et al. (2016) argue that the influence of language ability on symbolic play could possibly diminish with age. In our sample, age was not associated with the spontaneous interactions, nor did it meaningfully influence the correlations for the ADOS-2 Module or the VABS-2 CD on the interaction types when we partialled out age. Play and language are believed to follow similar developmental trajectories and build on shared skills, such as representational skills (Lifter et al., 2011). Our finding that the spontaneous interactions of autistic children with a robot is associated with their language ability is therefore in line with this belief. Note that most of the observed manifestations classified as functional interactions did not require the children to be able to use expressive language, and therefore does not explain the association with language ability.

The autism spectrum-related symptom severity and the child's social functioning followed a similar trend to language ability in their association with the interaction types, but correlated less strongly in our sample. The moderate to strong correlations between the child's language ability, autism-spectrum related symptom severity, and social functioning, may be one explanation for finding a trend similar to that of language ability, as it suggest that they measure something similar, such as their developmental level. Indeed, as the developmental level increases, autistic children start engaging in more complex types of object play (Vig, 2007; Naber et al., 2008; Thiemann-Bourque et al., 2012). Even though robots may be seen as social actors rather than objects, it may be that autistic children similarly engage in more complex interactions with the robot as their developmental level increases. This could also provide an explanation why we found no evidence of a relation between the child's chronological age and the interaction types, as chronological age is not a good indicator for the developmental level of an autistic child due to the developmental nature of ASC. Unfortunately, there was no measure available for our sample for assessing the relation of developmental levels on the spontaneous interactions in a robot-assisted intervention setting.

We argued previously that the ability to initiate may lead to more learning opportunities. In our sample, we found positive associations with the total number of spontaneous interactions with language ability, and social functioning. In like manner, a case study conducted by Duquette et al. (2008) found that non-verbal autistic children seemed less interested and engaged in human-mediated or robot-mediated sessions than pre-verbal autistic children. For the purpose of improving social initiation skills, it may be that it could be particularly beneficial to autistic children with limited language ability, but may also more difficult to achieve, as our results seem to indicate. Adding technology, such as a robot, may potentially serve as a scaffolding tool by providing an interesting, yet less complex, manner of social interaction. However, robot-assisted interventions that target different skills and are designed to capitalize on the robot's ability to elicit social interactions to another person may be less successful for these children as the robot may often fail to elicit such interactions.

### 5.3. Limitations

In this study, we interpreted the autistic children's spontaneous interactions as interactions for which they were motivated. One of the criteria for the annotations was that the child's initiation was visibly unprompted. However, with this method it is not possible to exclude interactions that were prompted in previous interactions in the robot-assisted intervention. Additionally, while the children were motivated to initiate the unprompted interactions, the nature of the motivations may differ. The children may have initiated an interaction for the sole purpose of having that interaction (they were intrinsically motivated), or to achieve another purpose (they were extrinsically motivated). For example, we observed that some of the school's staff members who were present during the session would encourage the child to say hello and goodbye. Such instances were not annotated, as they are prompted, but it may be that other children spontaneously said hello or goodbye because of similar rote learning. Their motivation may have been to adhere to a social norm, or to avoid a reminder to say hello and goodbye.

As a descriptive study, we did not select participants to answer our research question, and instead used an existing database of autistic child-robot interactions. The children featured in the DE-ENIGMA database are autistic children with no expressive speech up to the use simple phrases, many of whom had additional intellectual disabilities and language challenges. This limits our results to this specific subset of the autism spectrum, and does not necessarily generalize to autistic toddlers or autistic children with fluent expressive speech, who would be assessed with the ADOS-2 Module 3. While we looked at one of the largest samples of autistic children interacting with a robot, there may be other factors that influence whether and what type of spontaneous interactions they engage in, such as cultural differences (Rudovic et al., 2017). Additionally, not all interaction types were supported through the design of the intervention used in the DE-ENIGMA database, or through the protocol that was used by the adults. This affected exploratory and relational interactions in particular, as the exploratory interactions were often discouraged, and the relational interactions require the presence of additional objects. Also, the learning content of each session was different, which could have influenced the types of interaction that were observed. Future studies with different ASC are required to further investigate what individual factors influence the type of interaction autistic children spontaneously engage in, so that we may better tailor the robot's behavior to the children's needs and interests.

Some children noticed that there was a keypad, and that pressing buttons on that keypad would result in the robot performing certain actions. From such actions, it is possible that they subsequently derived that the robot was in fact being controlled by the adult, which then could have influenced the agency they attributed to the robot. Given that most of the children had additional intellectual disabilities, it is uncertain whether they would be able to infer that the robot was being controlled by the adult, nor whether they considered this to be the most plausible explanation for the keypad's function. Moreover, as we mentioned in section 2.1, it unclear to what extent autistic children consider a robot as a social actor to begin with.

Lastly, the autistic children in the DE-ENIGMA database interacted with a humanoid robot, with specific morphological and behavioral features. Robots with different morphology's and behaviors afford different types of interaction (Feil-Seifer and Matarić, 2011a). Therefore, the type of interactions initiated by the autistic children, and frequency thereof, may vary with other robots of a different morphology.

## 6. Conclusion

In this descriptive study, we investigated what types of interaction autistic children spontaneously engage in within a robot-assisted intervention setting, and how these types of interaction relate to the individual characteristics of the autistic children. We frequently observed autistic children spontaneously engaging in exploratory and functional interactions with the robot, and robot-elicited interaction between the child and adult. In particular, autistic children with stronger language ability, social functioning, and fewer autism spectrum-related symptoms, initiated more functional interactions with the robot and robot-elicited interactions with the adult. None of the individual characteristics were associated with the initiations of exploratory interaction with the robot.

To promote the engagement of autistic children to a robot-assisted intervention, we conclude that certain types of interaction may work better than other interaction types depending on the child's autism spectrum-specific characteristics. Facilitating learning through a specific interaction type, coupled with providing more autonomy over the robot's behavior to the autistic children, may enable them to stay engaged longer, facilitate more learning opportunities, and ultimately improve the effectiveness of a robot-assisted intervention. Our results indicate that the child's language ability may prove a useful heuristic in predicting what type of interaction with the robot can be motivating to the child. To this end, other ASC diagnostic assessments may also be insightful, but were so to a lesser degree in our sample. Facilitating a certain type of interaction will also affect the choice for a robot platform as it should support the facilitation of certain interaction types. In particular, exploratory interactions through touch are currently problematic for many robot platforms, as it can easily damage the robot. Our results indicate that such interactions with the robot are likely for autistic children, and should therefore be facilitated by the robot for it to become usable in practice. The differences among autistic children in their interaction within a robot-assisted intervention also underline the importance of reporting on autism-specific child characteristics to be able to generalize to other autistic children, which is currently not always the case (Begum et al., 2016).

The results of our study provide promising avenues for the design of deliberate robot behavior to keep autistic children engaged in a robot-assisted intervention and to account for the heterogeneity of these children. Future studies are required to translate our finding into interaction design for certain robot platform and assess whether they elicit and maintain the desired interaction. Experimental research is required to draw more firm conclusions whether designing for certain interaction types, or having child-led interactions in a robot-assisted intervention actually improves engagement and provide autistic children with more learning opportunities.

## Data Availability Statement

The DE-ENIGMA multi-modal database of autistic children's interactions used for this study is available to academic researchers worldwide. The database can be found on can be found here: https://deenigmadb.wordpress.com/. Note that no ethical approval was obtained for sharing the children's individual characteristic measures used for this study with others outside the DE-ENIGMA consortium. These measures are therefore not included in the DE-ENIGMA database, nor can they be made available on request.

## Ethics Statement

The data analyzed in this study was collected for the DE-ENIGMA database. The data collection study that led to the DE-ENIGMA database involved human participants and was reviewed and approved by the University College London, Institute of Education (REC 796). Written informed consent from the participants' legal guardian/next of kin was obtained to participate in the data collection study that led to the DE-ENIGMA database. As this study analyzes already collected data, ethical review and approval was not required in accordance with the local legislation and institutional requirements.

## Author Contributions

BS participated in the data collection for the DE-ENIGMA database, conducted the analysis, and wrote the manuscript. This process was supervised by DR, DH, and VE. All authors provided the feedback and guidance on all aspects of this study and its writing.

### Conflict of Interest

The authors declare that the research was conducted in the absence of any commercial or financial relationships that could be construed as a potential conflict of interest.

## Footnotes

^1^Paper in preparation. See the Data Availability Statement for more information on accessing the database.

^2^https://tla.mpi.nl/tools/tla-tools/elan/
